# A Study of Factors Affecting Functional Outcomes in Patients With Successful Recanalization by Mechanical Thrombectomy

**DOI:** 10.7759/cureus.54085

**Published:** 2024-02-12

**Authors:** Ichiro Deguchi, Takashi Osada, Toru Nakagami, Shinya Kohyama, Shinichi Takahashi

**Affiliations:** 1 Departments of Neurology and Cerebrovascular Medicine, Saitama Medical University International Medical Center, Hidaka, JPN; 2 Department of Endovascular Neurosurgery, Saitama Medical University International Medical Center, Hidaka, JPN

**Keywords:** white matter lesions, treatment outcome, thrombectomy, neurologic symptoms, ischemic stroke

## Abstract

Background and purpose: Reperfusion therapy is typically performed in cases with acute cerebral infarction. Mechanical thrombectomy (MT) achieves superior recanalization and favorable outcomes. However, some patients have poor functional prognosis despite successful recanalization. We investigated factors affecting functional prognosis after MT with good reperfusion.

Methods: Among the 205 consecutive cases with ischemic stroke treated with MT at our center from January 1, 2019 to March 31, 2021, 168 with successful recanalization were included. Factors affecting early neurological improvement (ENI) and modified Rankin Scale (mRS) scores were reviewed retrospectively.

Results: There were 93 (55%) cases with ENI and 75 (45%) without ENI. The times from onset to recombinant tissue-type plasminogen activator administration and recanalization in ENI cases were shorter than those in non-ENI cases. However, non-ENI cases had significantly higher Fazekas grades for white matter lesions. In multivariate analysis, the Fazekas grade was related to ENI (odds ratio [OR]=0.572, 95% confidence interval [CI]=0.345-0.948). The mRS score at discharge was 0-2 in 64 cases (good outcome) and 3-6 in 104 cases (poor outcome). Patients with a poor outcome had a significantly higher age, National Institutes of Health Stroke Scale (NIHSS) score, and Fazekas grade. Multivariate analysis revealed that the NIHSS score (OR=1.073, 95% CI=1.020-1.129) and Fazekas grade (OR=2.162, 95% CI=1.458-3.205) at hospitalization affected the mRS score at discharge.

Conclusion: There is a correlation of greater severity of white matter lesions with poorer ENI and clinical outcomes at discharge post-MT.

## Introduction

Mechanical thrombectomy (MT) can improve neurological symptoms in patients with ischemic stroke. MT has a very high recanalization rate and results in good outcomes, as evidenced by several large clinical studies [1-5]. However, a subset of patients fails to achieve favorable functional prognosis despite good recanalization with MT [6]. Based on previous reports, factors other than recanalization that affect functional prognosis after MT include age, pre-treatment National Institutes of Health Stroke Scale (NIHSS) score, recombinant tissue-type plasminogen activator (rt-PA) therapy, antithrombotic drugs, infarct size, collateral blood flow, blood glucose level, white matter lesions, and time to reperfusion [6-9]. However, factors affecting functional prognosis restricted to patients with good reperfusion have not been widely studied [6]. Therefore, this study retrospectively examined the factors that are associated with a failure to achieve a favorable functional prognosis despite good reperfusion by MT.

## Materials and methods

The study included 168 patients with successful recanalization (modified treatment in cerebral ischemia (mTICI) scale: grade 2b or 3) [10] out of 205 consecutive cases of ischemic stroke who were independent in activities of daily living prior to hospitalization (modified Rankin scale (mRS) score of ≤1) [11] and underwent MT (with/without rt-PA) at our hospital from January 1, 2019 to March 31, 2021. Patients were retrospectively evaluated for factors affecting early neurological recovery and clinical outcomes. We considered the following items: age, sex, cerebral infarction risk factors (hypertension, diabetes mellitus, hyperlipidemia, smoking [current smoker], and atrial fibrillation), previous ischemic stroke, history of coronary artery disease, body weight (BW), body mass index (BMI), systolic and diastolic blood pressure on admission, laboratory data on admission (blood glucose and creatinine clearance [CCr] from the Cockcroft-Gault equation) [12], NIHSS scores (at admission and 24 h after MT) [13], use of antithrombotic drugs at the time of stroke onset, type of cerebral infarction, size of infarct area (Alberta Stroke Program Early Computed Tomography Score on diffusion-weighted imaging [DWI-ASPECTS])[14], white matter lesions, use of general anesthesia, number of rt-PA therapies, time from onset to rt-PA administration, time from onset to reperfusion, large-vessel occlusion determined using magnetic resonance angiography, collateral circulation, symptomatic intracerebral hemorrhage (sICH), and clinical outcomes at hospital discharge.

Cerebral infarction was classified using the Trial of ORG 10172 in Acute Stroke Treatment criteria [15]. Early neurological improvement (ENI) was defined as a reduction in NIHSS score of >8 points or an NIHSS score of 0 or 1 at 24 h post-MT [3]. Clinical outcomes were evaluated using the mRS score [11]. Fluid-attenuated inversion recovery (FLAIR) images obtained at admission were used to classify the severity of white matter lesions into grades 0-3 (0=absence, 1=punctate foci, 2=beginning confluence, 3=large confluent areas of deep white matter hyperintense (DWMH) signals), as proposed by Fazekas to reflect deep and subcortical white matter lesions [16]. The lesions were graded by two neurologists (I.D., T.O.). The presence or absence of collateral circulation was determined by a physician (T.N.) familiar with angiography but blinded to the MT outcome when reviewing the angiographic images taken during MT treatment.

Intracranial hemorrhage on CT or MRI within 36 h post-MT, along with a reduction of the NIHSS score by ≥4 points, was taken to indicate sICH [17].

This retrospective study was approved by the Human Research Ethics Committee of Saitama Medical University International Medical Center (No.2022-102) and conducted by the opt-out method using our hospital website.

Statistical analysis

Data were analyzed with IBM SPSS Statistics for Windows, Version 20 (Released 2011; IBM Corp., Armonk, New York, United States). Age, systolic and diastolic blood pressure, BMI, BW, blood glucose, CCr, time from onset to rt-PA therapy, and time from onset to recanalization were compared between groups using Student's t-test; NIHSS score, DWI-ASPECTS, and DWMH grade were compared by the Mann-Whitney U test; ratios were compared using the Fisher exact test (two-sided). Multiple logistic regression analysis was used to evaluate factors affecting dependent variables, with NIHSS score (ENI or non-ENI) and mRS score (good or poor outcomes) at discharge as dependent variables and patient characteristics with a significant difference as independent variables. P < 0.05 indicated significance in all analyses.

## Results

There were 93 (55%) cases of ENI and 75 (45%) of non-ENI. A comparison of patient backgrounds between the ENI and non-ENI cases is presented in Table 1. There were shorter times from onset to rt-PA administration and onset to recanalization in ENI cases, whereas blood glucose and Fazekas grades were significantly higher in non-ENI cases. There were no statistical differences in age, sex, type of cerebral infarction, DWI-ASPECTS, NIHSS score on admission, percentage of collateral blood flow, and site of large vessel occlusion between the two groups. Using multiple logistic regression analysis, Fazekas grade was related to ENI (odds ratio [OR]=0.572; 95% confidence interval [CI]=0.345-0.948) (Table 2). The proportion of patients with good (mRS: 0-2) and poor (mRS: 3-6) outcomes at discharge was 38% (n=64) and 62% (n=104), respectively. A comparison of patient background between the good and poor outcome groups is summarized in Table 3. The CCr was significantly higher in the good outcome group than in the poor outcome group, whereas age, NIHSS score on admission, and Fazekas grade were significantly higher in the poor outcome group. There were no statistical differences in sex, type of cerebral infarction, DWI-ASPECTS, percentage of collateral blood flow, time from onset to rt-PA administration, time from onset to recanalization, and site of large vessel occlusion were not statistically different between the two groups. Using multiple logistic regression analysis, the NIHSS score on admission and Fazekas grade were factors influencing outcomes at discharge (NIHSS score: OR=1.073, 95% CI=1.020-1.129; Fazekas grade: OR=2.162, 95% CI=1.458-3.205) (Table 4). The rate of sICH was significantly lower in cases of good outcomes compared with those of poor outcomes (0% versus 8%, P=0.025).

**Table 1:** Clinical background of ENI and non-ENI cases Data are shown as mean ± standard deviation, median (interquartile range), or number (%). BA, basilar artery; DWI-ASPECTS, Alberta stroke program early computed tomography score on diffusion-weighted imaging; ENI, early neurological improvement; ICA, internal carotid artery; MCA, middle cerebral artery; NIHSS, National Institutes of Health Stroke Scale; rt-PA, recombinant tissue plasminogen activator; mTICI, modified treatment in cerebral ischemia. *P<0.05.

Variable	ENI (n=93)	Non-ENI (n=75)	P-value
Age, years	73.6±10.8	75.4±11.2	0.293
Female sex	31 (33)	24 (32)	0.870
Hypertension	58 (62)	43 (57)	0.530
Diabetes mellitus	18 (19)	24 (32)	0.073
Hyperlipidemia	47 (51)	33 (44)	0.439
Atrial fibrillation	59 (63)	45 (60)	0.749
Coronary heart disease	10 (11)	11 (15)	0.487
Cerebral infarction	19 (20)	8 (11)	0.096
Smoking	35 (38)	26 (35)	0.748
Systolic blood pressure, mmHg	160.7±28.3	167.4±29.8	0.141
Diastolic blood pressure, mmHg	87.2±22.9	89.0±26.6	0.636
Body mass index, kg/m^2^	21.5±3.2	22.5±4.1	0.073
Body weight, kg	56.1±10.7	58.7±12.8	0.157
Glucose level, mg/dL	134.0±57.6	158.0±70.6	0.017*
Creatinine clearance, mL/min	62.3±26.9	65.4±31.8	0.494
NIHSS score on admission	18 (14–22)	15 (9–22.5)	0.067
Treatment with antithrombotic drugs on admission	33 (35)	25 (33)	0.871
Stroke subtype			
Cardioembolism	60 (64)	47 (63)	0.872
Large-artery atherosclerosis	25 (27)	22 (29)	0.728
Undetermined etiology	8 (9)	6 (8)	1.000
DWI-ASPECTS	7 (6–9)	7 (6–8)	0.844
Fazekas scale grade	1 (0–2)	2 (1–3)	<0.001*
rt-PA	37 (40)	28 (37)	0.753
Onset to rt-PA, min	137.7±76.0	196.7±140.6	0.033*
Onset to recanalization, min	285.1±193.2	381.3±254.8	0.006*
mTICI grade 3	56 (60)	37 (49)	0.164
Collateral circulation	47 (51)	45 (60)	0.275
General anesthesia	3 (3)	3 (4)	1.000
Large vessel occlusion			
ICA	40 (44)	23 (31)	0.109
MCA-M1	43 (47)	37 (49)	0.758
MCA-M2	4 (4)	8 (11)	0.139
BA	5 (5)	7 (9)	0.378

**Table 2:** Factors affecting ENI (multiple logistic regression analysis) CI, confidence interval; ENI, early neurological improvement; rt-PA, recombinant tissue plasminogen activator. P<0.05.

	Odds ratio	95% CI	P-value
Glucose level	0.999	0.991–1.005	0.726
Fazekas scale	0.572	0.340–0.948	0.030*
Onset to rt-PA	0.995	0.983–1.007	0.415
Onset to recanalization	0.999	0.989–1.009	0.804

**Table 3:** Clinical background of cases of good (mRS 0–2) and poor (mRS 3–6) outcomes Data are shown as mean ± standard deviation, median (interquartile range), or number (%). BA, basilar artery; DWI-ASPECTS, Alberta stroke program early computed tomography score on diffusion-weighted imaging; ENI, early neurological improvement; ICA, internal carotid artery-occlusion; MCA, middle cerebral artery; mRS, modified Rankin Scale; NIHSS, National Institutes of Health Stroke Scale; rt-PA, recombinant tissue plasminogen activator; mTICI, modified treatment in cerebral ischemia. *P<0.05.

Variable	Good outcome (n=64)	Poor outcome (n=104)	P-value
Age, years	70.4±12.3	76.8±9.4	<0.001*
Female sex	17 (27)	38 (37)	0.236
Hypertension	39 (61)	62 (60)	1.000
Diabetes mellitus	13 (20)	29 (28)	0.359
Hyperlipidemia	31 (48)	49 (47)	0.875
Atrial fibrillation	38 (59)	66 (63)	0.626
Coronary heart disease	9 (14)	12 (12)	0.638
Cerebral infarction	12 (19)	15 (14)	0.519
Smoking	26 (41)	35 (34)	0.410
Systolic blood pressure, mmHg	162.7 ± 28.6	165.8 ± 32.9	0.531
Diastolic blood pressure, mmHg	84.8 ± 18.9	90.0 ± 27.4	0.184
Body mass index, kg/m^2^	22.0 ± 3.7	21.9 ± 3.5	0.814
Body weight, kg	58.6 ± 12.6	56.4 ± 11.1	0.252
Glucose level, mg/dL	129.0 ± 29.4	144.0 ± 62.5	0.063
Creatinine clearance, mL/min	72.5 ± 32.8	58.2 ± 25.3	0.002*
NIHSS score on admission	14 (8–21)	19 (14–24)	<0.001*
Treatment with antithrombotic drugs on admission	24 (38)	34 (33)	0.616
Stroke subtype			
Cardioembolism	41 (64)	66 (63)	1.000
Large-artery atherosclerosis	17 (27)	30 (29)	0.860
Undetermined etiology	6 (9)	8 (8)	0.776
DWI-ASPECTS	8 (6–9)	7 (6–8)	0.139
Fazekas scale grade	1 (0–1)	2 (1–3)	<0.001*
rt-PA	29 (45)	36 (35)	0.193
Onset to rt-PA, min	176.9±142.3	153.2±81.9	0.324
Onset to recanalization, min	293.4±209.0	349.4±236.2	0.121
mTICI grade 3	33 (52)	60 (58)	0.523
Collateral circulation	34 (53)	58 (56)	0.752
General anesthesia	2 (3)	4 (4)	1.000
Large vessel occlusion			
ICA	22 (34)	41 (39)	0.515
MCA-M1	33 (52)	47 (46)	0.525
MCA-M2	7 (11)	5 (5)	0.216
BA	2 (3)	10 (10)	0.133

**Table 4:** Factors affecting the mRS score (multiple logistic regression analysis) CI, confidence interval; mRS, modified Rankin Scale; NIHSS, National Institutes of Health Stroke Scale. *P<0.05.

	Odds ratio	95% CI	P-value
Age	1.020	0.972–1.070	0.421
Creatinine clearance level	1.001	0.984–1.019	0.879
NIHSS score on admission	1.073	1.020–1.129	0.006*
Fazekas scale	2.162	1.458–3.205	<0.001*

## Discussion

This study showed that ENI after MT and clinical outcomes at discharge were poor in cases with severe white matter lesions, even after successful recanalization with MT. Moreover, greater stroke severity before MT was associated with poorer clinical outcomes at discharge. Previous studies have reported that low NIHSS scores before MT were associated with good outcomes [7,18]. Consistent with these findings, the NIHSS score was found to be associated with the clinical outcomes of MT. Regarding white matter lesions, multiple reports have documented that greater severity of these lesions is related to poorer MT outcomes and associated with futile recanalization [9,19-22]. Conversely, several studies have reported that white matter lesions do not affect outcomes, while others have indicated that good outcomes can be obtained in some patients, even in the presence of severe white matter lesions [22-24]. Mistry et al. examined patients with successful recanalization and reported that outcomes at day 90 were worse in cases with severe white matter lesions than in those with mild lesions; however, in contrast to our findings, there was no difference in ENI between the two groups [20]. We previously found a poorer functional prognosis after MT in elderly patients compared to that in younger patients and highlighted the mechanistic involvement of deep white matter lesions [25]. Whether white matter lesions influence functional prognosis after MT, including the underlying mechanisms, has not been fully elucidated, but we speculate on the following mechanisms.　

**Figure 1:**
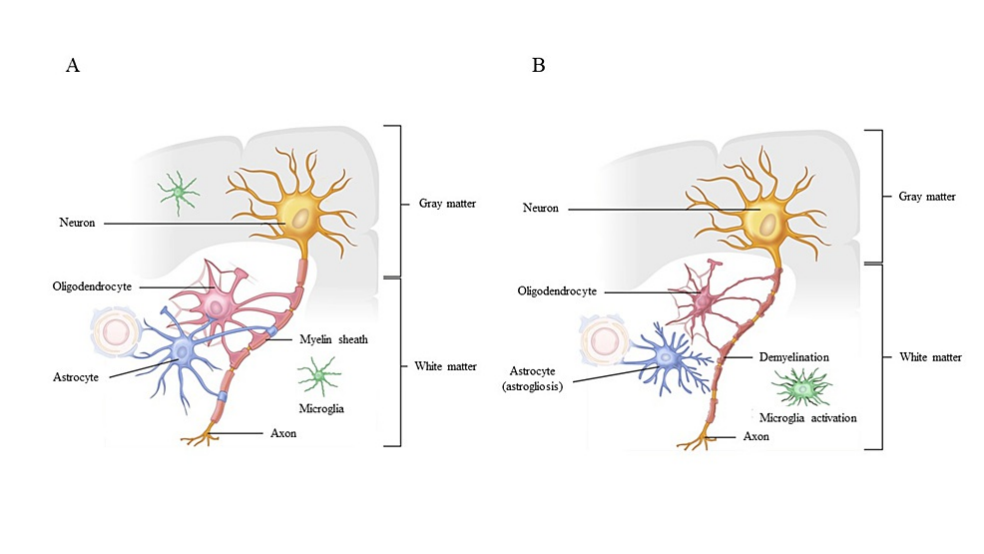
Normal white matter (A) and asymptomatic white matter lesions (B) (A) Anatomically, the myelinated axons and glial cells (astrocytes, oligodendrocytes and, microglia) are morphologically and functionally coupled, constituting the cerebral white matter (WM). Together, these are responsible for the normal appearance of the WM. (B) In patients with WM lesions, recovery from neurological symptoms is delayed compared with patients without WM lesions owing to underlying dysfunction of the glial cells, such as oligodendrocytes and astrocytes, that make up the WM． Image Credits: Ichiro Deguchi and Shinichi Takahashi (Authors)

Anatomically, axons and glial cells are morphologically and functionally coupled, constituting the cerebral white matter. On the other hand, in patients with white matter lesions, in the process of recovery from the sudden ischemic burden due to major artery occlusion, recovery of neurological symptoms (i.e., resilience) is delayed compared with patients without white matter lesions due to the underlying dysfunction of glial cells, including oligodendrocytes and astrocytes that support energy metabolism of axons [25-27] (Figure 1). Therefore, when endovascular treatment is performed in patients with acute stroke who have severe white matter lesions on presentation, it is important not only to achieve good recanalization but also to recognize that their brain is less tolerant and resilient to ischemia and to make a conscious effort to shorten the time from onset to reperfusion to restore neurological symptoms. In addition, white matter lesions are thought to be associated with platelet activation and hypercoagulability, leading to a "no-reflow" phenomenon in the focal area [20]. Therefore, it seems to be conceivable that white matter lesions are more likely to be larger infarcts despite recanalization.

The rate of sICH cases was higher in patients with poor outcomes in the current study. Six of the eight sICH cases had a Fazekas scale of 0-1 and mild white matter lesions. Factors associated with sICH were not examined, but the absence of an association of sICH after MT with white matter lesions has previously been suggested [9,20,21,23-25].

The study has some limitations, including its retrospective design and small number of cases. However, our findings contribute to a deeper understanding of neurological symptoms occurring at an early stage after MT and factors affecting clinical outcomes in patients with successful reperfusion. Further accumulation of cases and investigation of factors affecting early neurological symptoms and clinical outcomes, including elucidation of the involvement and underlying mechanisms of white matter lesions, are warranted.

## Conclusions

In conclusion, early neurological symptom improvement after MT and clinical outcomes at discharge were poor in cases with severe white matter lesions, despite successful recanalization with MT. Therefore, when endovascular treatment is performed for acute stroke in patients with severe white matter lesions on presentation, achieving good recanalization is necessarily important. However, recognizing that patients with severe white matter lesions have low tolerance and resilience to ischemia in the brain is also important to make conscious efforts to shorten the onset-to-reperfusion time for restoring neurological symptoms.
